# A Hybrid Feature Pool-Based Emotional Stress State Detection Algorithm Using EEG Signals

**DOI:** 10.3390/brainsci9120376

**Published:** 2019-12-13

**Authors:** Md Junayed Hasan, Jong-Myon Kim

**Affiliations:** Department of Electrical, Electronics and Computer Engineering, University of Ulsan, Ulsan 44610, Korea; junhasan@gmail.com

**Keywords:** EEG signals, stress analysis, feature selector, k-NN

## Abstract

Human stress analysis using electroencephalogram (EEG) signals requires a detailed and domain-specific information pool to develop an effective machine learning model. In this study, a multi-domain hybrid feature pool is designed to identify most of the important information from the signal. The hybrid feature pool contains features from two types of analysis: (a) statistical parametric analysis from the time domain, and (b) wavelet-based bandwidth specific feature analysis from the time-frequency domain. Then, a wrapper-based feature selector, Boruta, is applied for ranking all the relevant features from that feature pool instead of considering only the non-redundant features. Finally, the k-nearest neighbor (k-NN) algorithm is used for final classification. The proposed model yields an overall accuracy of 73.38% for the total considered dataset. To validate the performance of the proposed model and highlight the necessity of designing a hybrid feature pool, the model was compared to non-linear dimensionality reduction techniques, as well as those without feature ranking.

## 1. Introduction

For industrial safety, identifying risks from human error is necessary because unsafe and reckless behaviors of industrial workers and lack of precautions are directly responsible for human-caused problems. Some of the key factors of these unsafe and reckless behaviors include lack of proper sleep, lack of a proper diet, physical defects, and fatigue, which can lead a person into a stressful situation. This situation causes discomfort, anxiety, depression, cardiovascular disease, high heart rate, and several other harmful effects [1,2]. In general, stress is the body’s response to mental and physical pain. However, in a more scientific way, stress can be defined as a complex psycho-physiological state initiated by the discrepancy between the person’s perceived exogenous and endogenous demands (stressors) and its perceived competence to cope with these demands [3,4].

In recent years, techniques including functional magnetic resonance imaging (fMRI), near-infrared spectroscopy (NIRS), electrocorticography (ECoG), and, electroencephalogram (EEG) signals have been used to detect and analyze emotional states [5,6]. fMRI and NIRS measure brain activations using brain blood. fMRI has the benefit of determining signals inside the brain with an exceptional altitudinal resolution, but the measurements are deferred until the state of the brain changes. In contrast, NIRS can only elucidate the condition of the brain exterior, and the signal is ultimately acquired through blood flow. The ECoG and EEG signals quantify the brain waves. Despite ECoG having the great advantage of measuring long-bandwidth signals, electrode positioning on the shell of the brain to acquire the signals necessitates a surgical procedure. Alternatively, EEG uses a procedure that requires wearing a helmet [7], and therefore EEG can be measured non-invasively. It measures signals from the scalp rather than the brain itself [8]. Therefore, in this study, EEG is considered. The main purpose of this paper is to identify the mental state of a person by analyzing the EEG signals. 

Several studies have demonstrated the correlations between EEG pattern and emotional states, i.e., calmness, depression, excitement [9,10,11]. In [10], an EEG-based analysis on the frontal alpha asymmetry index with a support vector machine (SVM) algorithm was proposed for stress analysis. In [11], a frequency domain-based analysis with a k-nearest neighbor (k-NN) algorithm was proposed to analyze the stress state using the EEG signal. Recently, deep learning-based approaches have also been applied to classify different mental states by analyzing EEG signals. These methods automatically learn feature representation from the data to distinguish among different classes. Li et al. [12] used a spatial and temporal deep learning architecture to learn discriminative spatial-temporal EEG features for the detection of emotional states. Hefron et al. [13] suggested a novel convolutional recurrent neural model by using multipath subnetworks for a cross-participant EEG-based assessment of cognitive workloads. Here, the bi-directional-residual recurrent layers statistically signify the increment of performance in predictive accuracy. Kuanar et al. [14] designed an EEG-based multispectral time-series imaging technique with a recurrent neural network algorithm to do the cognitive analysis of working memory load. However, these deep networks usually need huge amounts of data for training purposes. In addition, deep features (i.e., the output from intermediate layers of deep networks) may be correlated and non-separable. Moreover, the existing approaches do not give any importance to the selection of suitable features from particular domains for deeper causal analysis.

In this study, an EEG data-driven mental state identification technique is developed to analyze whether a person is experiencing stress. The pre-processed signals from the Database for Emotion Analysis using Physiological Signals (DEAP) are considered for analysis [15]. By analyzing these signals, a custom hybrid feature pool is designed, which consists of two types of features: (1) statistical features from the time domain, and (2) wavelet-based features from the time-frequency domain. Once the feature space is available, the next step is to select the reliable features. Feature selection has a great impact on improving the classifier performance. In the supervised machine learning approaches, the corrected class labels of input data are known, and the feature evaluation criteria is applied to extract valuable features from the given data. The feature selection process serves two key purposes: (1) selection of relevant features, and (2) reduction of feature dimension. The traditional feature selection algorithms usually select the non-redundant or relevant attributes from a single dataset only and do not consider the variations in input datasets. In simple words, it might happen that certain features may be non-redundant/relevant for a particular dataset and redundant/irrelevant for another dataset. To address this issue, a wrapper-based approach called Boruta is utilized, which randomly shuffles sub-datasets and selects the relevant features accordingly. Finally, the features processed by the Boruta technique are supplied to the k-NN algorithm for classification. To demonstrate the robustness of the proposed method, it is compared with two cases: (1) dimensionality reduction of the hybrid feature pool by principal component analysis (PCA) and then the use of k-NN for classification, and (2) consideration of all the features (i.e., without using the feature selection technique) and then applying k-NN for classification. The main contributions of this paper are summarized as follows: (1) a hybrid feature pool is designed by combining the statistical features from the time domain and wavelet-based EEG band-wise features from the time-frequency domain to effectively capture all the intrinsic information from the EEG signal despite artifacts, and (2) a wrapper-based feature selection mechanism, Boruta, is deployed to analyze all the important attributes of the hybrid feature pool.

The remainder of this paper is arranged as follows. Section 2 describes the publicly available dataset and the details of each step of the proposed methodology. Data arrangement and in-depth analysis of the experimental results are provided in Section 3. The limitations and the future aspects of this research are discussed into Section 4. Finally, Section 5 concludes this paper.

## 2. Methodology

**Figure 1:**
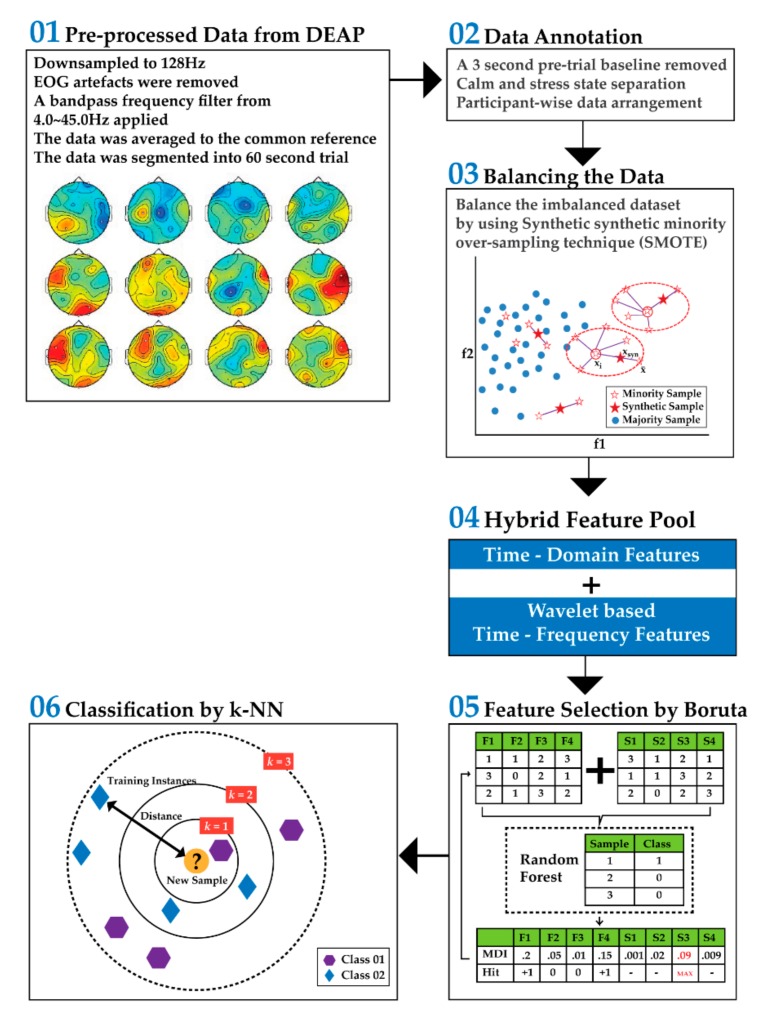
Block diagram of the proposed method [15].

In this study, the main goal is to identify signs of stress from EEG recordings. In Figure 1, a block diagram of the overall proposed method is given. The proposed approach is divided into five blocks: (1) pre-processed data collection from the DEAP dataset [15], (2) data annotation and arrangement, (3) creation of the hybrid feature pool, (4) discriminant feature selection by a wrapper-based feature ranked approach, Boruta, and (5) k-NN-based classification.

### 2.1. Dataset Description and Annotation

EEG signals from the DEAP dataset [15] are used for this mental stress classification task. This dataset comprises emotional responses induced by music videos. In total, 32 participants from the 19–37-year age group were tested to build this dataset. Each participant watched 40 music videos. While watching, EEG signals were recorded for 1 min with the 10–20 system of electrode placement. Each music video has a separate experiment ID. For each experiment ID, signals from 40 different channels were recorded. Among these 40 channels, 32 are EEG channels and 8 are peripherals. The specific details related to these channel descriptions are well documented in [15]. In this research, the main objective is to identify the calm or stress state of a person by analyzing the EEG signals. Verma et al. [16] compared the performance of 40 channel features (32 EEG and 8 peripherals) with that of 32 EEG channel features and observed that the 40-channel features did not bring any significant improvement over the 32 EEG channel features. For similar reasons, Zhang et al. [17] considered only EEG based feature extraction in identifying the emotional state of a person. Therefore, in this study, the 32 EEG channels are considered for the final analysis. 

After considering the 32 EEG channels, the valence and arousal levels are analyzed from the online self-assessment of each participant for each experiment ID [18,19]. Every experiment ID has a predefined online rating, by which all the experiment IDs can be categorized either the genre of stress state or the genre of calmness. However, when a participant provides the self-assessment rating for the same video, from the participant rating list (available on [15]), it can be observed that the video from the genre of calmness brings the feeling of stress and vice-versa. Therefore, for this research, the online self-assessment rating is considered to categorize the experiment IDs (either calm or stress) for each participant by Equations (1) and (2), derived from [11,20,21].

(1) calm=(4<valence<6)∩(arousal<4)

(2) stress=(valence<3)∩(arousal>5)

After differentiating the data into two states, 7 participants (participant numbers 3, 6, 7, 9, 17, 23, and 30) did not show any distinctive mental state of calm and stress. With the remaining 25 participants, the dataset was arranged for the classification task, which is depicted in Table 3 of Section 3.1. While arranging the dataset, some datasets depicted an imbalanced nature. To tackle the imbalanced nature of these datasets, the synthetic minority over-sampling technique (SMOTE) [22] was adopted before applying the feature design and classification steps.

### 2.2. Hybrid Feature Pool

The main objective of designing the hybrid feature pool is to obtain reliable information from EEG signals for emotional state identification. To form a reliable feature pool, analyzing the signals from different domain perspectives is necessary. Therefore, the hybrid feature-attributes are measured from two specific domain-based analyses: (a) statistical features from time-domain analysis, and (b) wavelet-based feature analysis from the time-frequency domain. In this study, a total of 19 features is considered, 11 from the time domain and 8 from the wavelet-based time-frequency domain.

### 2.3. Statistical Features from the Time Domain

From the time domain, the extracted statistical features are root mean square (F1), square mean root (F2), peak to peak (F3), kurtosis (F4), skewness (F5), kurtosis factor (F6), shape factor (F7), crest factor (F8), and impulse factor (F9). In addition to these statistical feature parameters, Hjorth parameters are also considered to compute the mobility and complexity of the signal [20,23,24]. These two parameters contain the information on the frequency spectrum of the signal. Mobility is F10 and complexity is F11. All 11 features are mathematically defined in Table 1.

### 2.4. Wavelet-Based Feature Analysis from the Time-Frequency Domain

**Figure 2:**
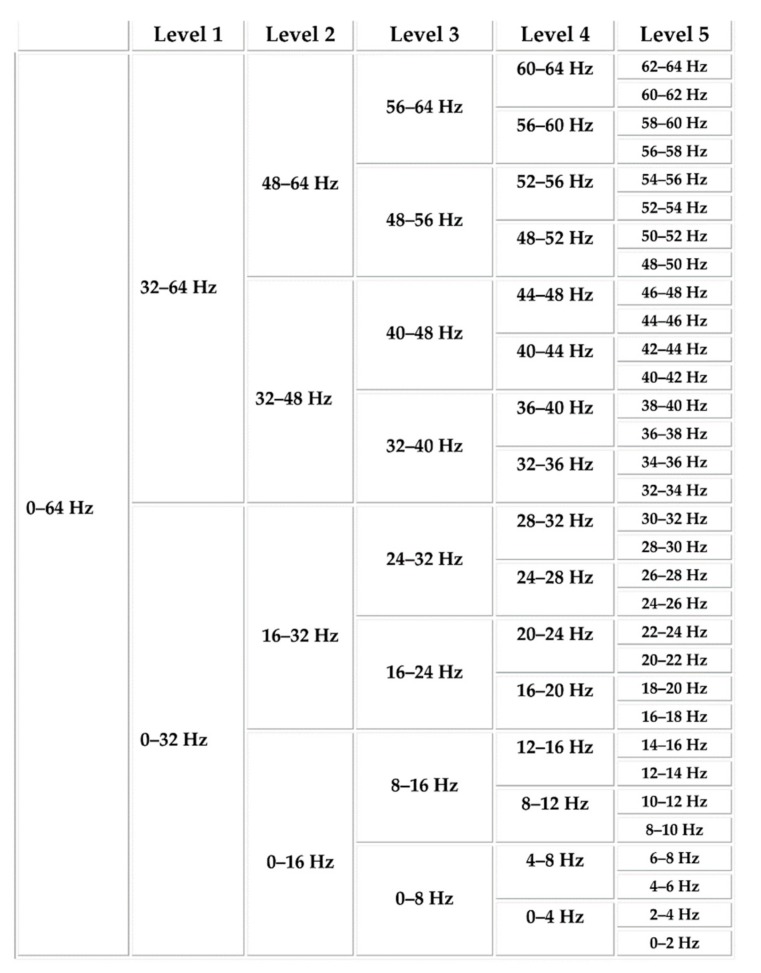
Decomposition tree of level 5 discrete wavelet transform (DWPT).

Usually, the EEG signal is divided into five distinct frequency bands of delta, theta, alpha, beta, and gamma [25,26]. In this study, the considered dataset is downsampled to 128 Hz, smearing a 4 to 45 Hz bandpass filter and eliminating EEG artifacts. So, the delta band (0–4Hz) is not present in the dataset for further analysis. However, from the present frequency bands of the signals, to extract and analyze the time-frequency based wavelet features, discrete wavelet transform (DWPT) is considered in this case. By considering the Daubechies 4 wavelet (db4) function, a level 5 DWPT is applied to the existing signals. The resulting DWPT decomposition tree is depicted in Figure 2.

The details of the frequency bands and considered correlated DWPT packets are provided in a very detailed manner in Table 2.

To obtain four distinct bandwidths, five DWPT packets are considered. Therefore, from the wavelet coefficient vector, two features are calculated: energy (F12) and standard deviation (F13). From each wavelet band, the entropy is calculated, and their sum is considered as a separate feature, denoted as the wavelet sum of entropy (F14). Then, from each DWPT packet, the power spectral density (PSD) is calculated by using the Welch method [27]. For the five DWPT packets, five power bands are calculated and considered as five separate features from F15 to F19.

### 2.5. Feature Selection by Boruta

As discussed in the introductory part, the objective of feature selection is to obtain the minimal-optimal feature set (i.e., the smallest possible feature set). The traditional feature selection algorithms rely upon classification accuracy to decide the importance of a feature. The features which generally improve classification accuracy are non-redundant features; otherwise they are redundant in nature. The removal of an important feature may decrease the classification accuracy; however, no significant change in the classification accuracy does not signify that the feature is irrelevant or unimportant. Thus, the selection of all relevant attributes instead of just the non-redundant ones is important for preventing the loss of any useful information from the feature set [28,29]. A wrapper method usually uses a classifier wrapped around the feature selection process for deciding all the important features. The Boruta algorithm adopted in this study is a wrapper-based technique built around random-forest (RF) [30] classifier. The algorithm consists of these following steps:

1. First of all, it duplicates the original features to extend the feature information. The duplicated attributes are known as shadow features.
2. Then it shuffles the attributes of those shadow features to remove their correlations with the response.
3. After that, it trains the shadow features around the random forest (RF) classifier to justify the importance of individual features by the mean decrease impurity (MDI) matrix. MDI calculates the number of times a feature is used to split a node, weighted by the number of samples it splits across all the trees of the RF classifier. Thus, MDI decides the importance of each shadow feature. The shadow feature with the highest MDI score is considered as the best shadow feature.
4. Now, the algorithm tests the real feature attributes to determine whether they are important. For this purpose, the Z score is needed. In tree-based machine learning algorithms (i.e., RF), the significance measure of a feature is attained as the loss of accuracy of classification caused by the random permutation of characteristic values between attributes. This loss is computed individually for all trees in the forest, which use a provided attribute for categorization. Then the average and standard deviation of the accuracy losses (from individual RF trees) are computed. The Z score is finally computed as dividing the average loss by its standard deviation [28]. In Boruta, the Z score is used as the importance measure since it considers the fluctuations of the mean accuracy loss among trees in the forest (RF classifier). Since the Z score cannot be used directly to measure the feature importance, some external references are needed to decide whether the importance of any given attribute is significant. Therefore, with the MDI score, the most significant shadow features are considered as the external reference to determine the important attributes from the original feature set. Thus, after the RF classifier is applied, the algorithm assesses whether any of the original feature attributes have a higher Z score (ZOF) than the Z score of that important shadow feature (MZSF). If the ZOF is higher than the MZSF, then the algorithm records this event as a count in a vector corresponding to the original features, called a “hit”. From this hit vector, the important feature set is obtained. The unimportant features are discarded.
5. The procedures from Step 1 to 4 are repeated until a significance is assigned for all the attributes, or the algorithm has attained the earlier set limit of the RF classifier runs (iteration limit) [28].

In short, Boruta is centered on a similar idea which structures the basis of the RF classifier. It combines randomness to the procedure and accumulate results from the ensemble of randomized samples. Especially, the significance of a shadow feature can be non-zero only due to these random fluctuations. Thus, the set of important shadow features is utilized as a character reference for determining which original features are truly essential. Therefore, it is necessary to repeat the re-shuffling technique for generating different shadow features each time to achieve the statistically reasonable scores. In this work, in total, 20 iterations are considered to serve this purpose.

### 2.6. Classification by the k-Nearest Neighbor (k-NN) Algorithm

**Figure 3:**
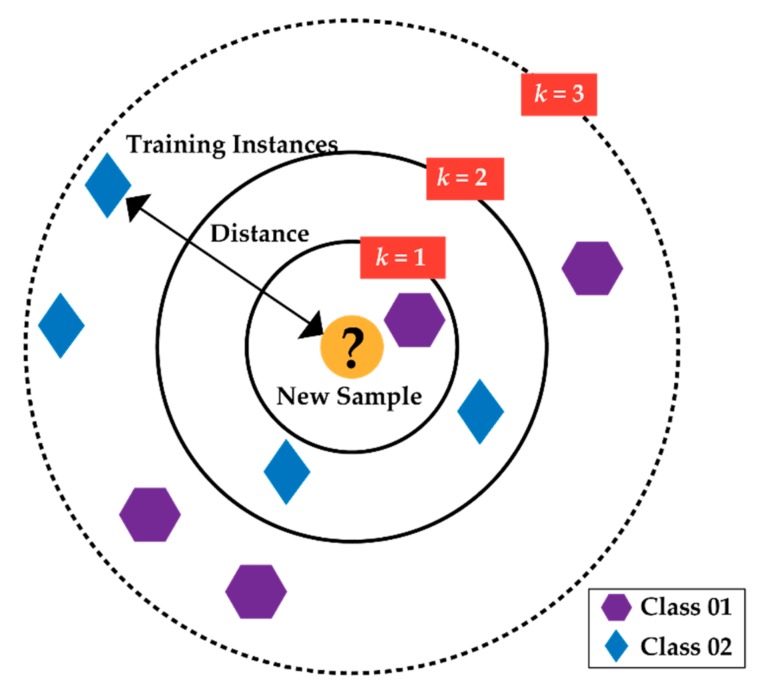
Descriptive illustration of k-NN algorithm.

The ranked feature subset is finally classified by the k-NN algorithm. The advantages of this are simplicity in building architecture and less computational complexity for small-sized data [31,32]. Because of non-parametric attitudes and classifying samples based on votes of k-nearest neighbors, it is efficient to use for a small featured dataset. This k-NN algorithm performs on three main principles: (a) calculates the distance between the neighbors, (b) finds the *k* closest neighbors to deal with the bias-variance trade-off for solving the overfitting/underfitting problem, and (c) votes for labels. From the illustration of Figure 3, the main mechanism of the k-NN algorithm can be easily explained.

As in Figure 3, the new data point (inside the circle, yellow color) can be categorized either as class 01 (purple polygon) or as class 02 (blue diamond). When *k =* 3, the new data point fits into class 02 because of the higher density of class 02 within the circle, i.e., there are two blue diamonds (class 02) and one purple polygon (class 01) within the second circle. If the value of *k* is randomly assigned, i.e., *k* = 5, then the data point fits into class 01 because three instances from class 01 and two instances from class 02 are surrounded by the outmost black dashed circle. So, the selection of the *k* value is important. With a given *k*-value, boundaries of each class can be drawn. These boundaries segregate class 01 from class 02. Therefore, there are two significant parameters that should be selected to establish the classification task of k-NN: (a) the optimal value of *k* that defines the number of neighbors and (b) the distance metric, which is calculated by the Euclidean distance from Equation (3).

(3) $$D_{x,y}=\sqrt{{\sum_{i=1}^{n}\left(x_{i}-y_{i}\right)}^{2}}$$

**Figure 4:**
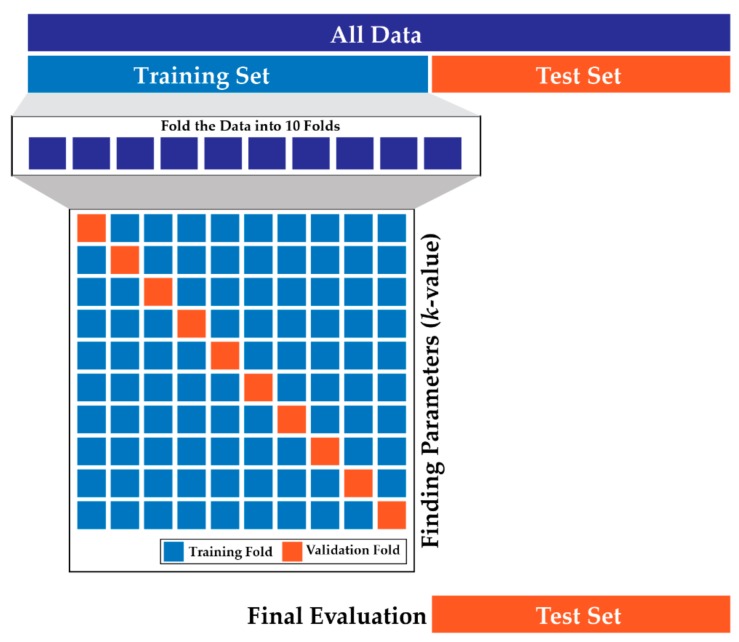
K-fold cross-validation process in k-NN for selecting the *k-*value.

To find the optimal value of *k*, the K-fold cross validation is used. As depicted in Figure 4, the K-fold cross-validation (the nearest-neighbor *k* is different from this K) involves randomly dividing the training set into 10 groups, or folds. After that, the training dataset is divided into two sets: training folds A, and validation fold B. The model is trained based on training folds A, and tested against the validation fold B. The validation fold B is used to tune the parameters, such as the *k* in k-NN. The validation fold is rotated in every iteration (10 times) and the rest of the data is used to train the k-NN. In simple words, a K-fold cross-validation implies splitting the data into K fold, then, training on (K–1) folds, and testing on the remaining 1-fold as the validation fold. 

In this research, each of the considered datasets (datasets 1 to 25 in Table 5, the merged dataset in Table 6) is first divided into training and testing sets at a 60/40 ratio. Then, a 10-fold cross-validation is performed on the test set of every dataset using a generated list of odd *k*s ranging from 1 to 20. On every iteration of 10-fold cross-validation, the misclassification error vs. *k* is observed. For most of the datasets, the optimal value for *k* ranges from 4 to 9 after a 10-fold cross-validation.

## 3. Result Analysis and Discussion

### 3.1. Dataset Arrangement

The considered DEAP dataset holds pre-processed data that downsample the recorded signals to 128 Hz, smearing a 4 to 45 Hz bandpass filter and eliminating EEG artifacts. In this paper, the pre-processed data with 128 Hz sampling frequency is considered. Each pre-processed signal is around 1 min 3 s in length. First, the 3-second pre-train baseline is removed from the pre-processed signal. Then, the remaining 1-minute signal is considered as the final arrangement. The data from each participant ID are considered as an individual dataset for conducting the experiment. The details of the dataset are given in Table 3.

Table 3 shows that each dataset has a unique experimental ID, which highlights the distinctive states. Therefore, for precise and logical analysis according to experimental ID, each participant is considered a separate dataset.

### 3.2. Performance Analysis of Feature Selection by Boruta from the Hybrid Feature Pool

**Figure 5:**
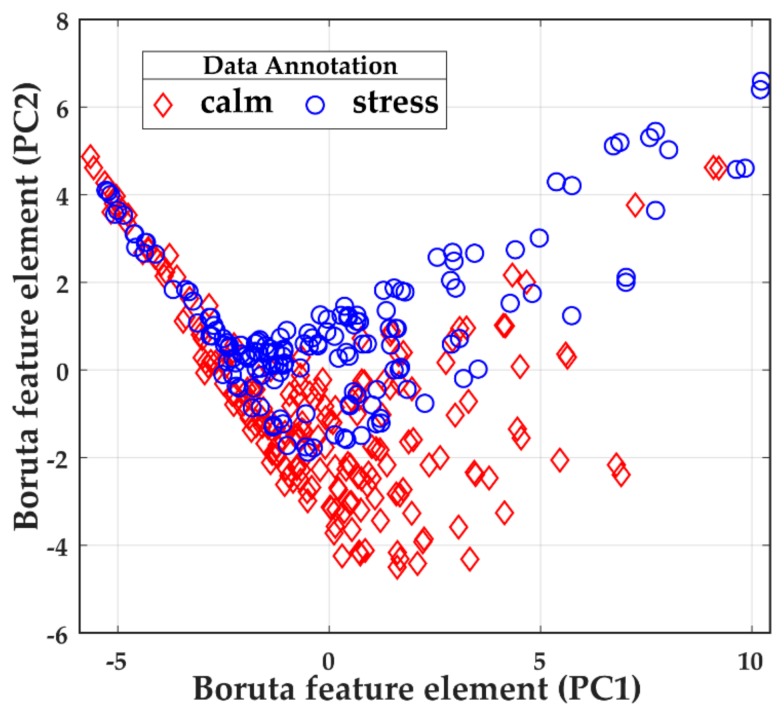
Boruta feature space for Dataset 2.

Table 3 shows that each participant has a distinctive mental state (calm and stress) for different experimental IDs. The Boruta provides an identical ranking of features for individual datasets. For example, for Dataset 1, Boruta ranks 9 of 19 features as the most meaningful, whereas for Dataset 3, it ranks 13 features as most important. This difference highlights the necessity of considering the datasets separately based on participant ID. Figure 5 shows features selected by Boruta for Dataset 2 in 2D feature space. For visualization purposes, the selected ten features are first compressed into 2D space by PCA (2 PC components values: PC1, and PC2) and then plotted in Figure 5. As shown in Figure 4, the Boruta features provide satisfactory separation for different types of emotional states in spite of the fact that the EEG data from different mental states are highly correlated with each other.

### 3.3. Performance Analysis of the k-Nearest Neighbor Algorithm

The optimal feature subset from each dataset is delivered to the k-NN for final classification. The dataset is divided into training and testing sets at a 60/40 ratio. Equation (4) determines the class-wise accuracy.

(4) $$Classwise\_accuracy=\frac{True\_positive}{True\_positive+false\_positive}$$

To determine the average classification accuracy, Equation (5) is used.

(5) $$Avg.\_accuracy=\frac{True\_positive+True\_negetive}{Total\_number\_of\_samples}$$

Table 3 shows that most of the considered datasets are imbalanced. For example, in Table 3, Dataset 1 exhibits the calm state for two experimental IDs (9 and 14) and the stress state for six experimental IDs (17, 32, 34, 35, 36, and 37). This is an extreme example of an imbalanced dataset from Table 2. To tackle this imbalance nature of these datasets, SMOTE [22] is adopted before calculating the final performance of the classifier (i.e., class-wise accuracy and average accuracy). The details of the class-wise accuracy along with average accuracy are given in Table 4. The classifier is tested for ten iterations with random values of the hyperparameters, and comparable results are observed in each iteration. The classification accuracies in various iterations deviate from each other within a negligible range of 0.2%–1%. 

### 3.4. Comparison Analysis

To establish the efficiency of the proposed method, two comparisons are made for each dataset. Two approaches other than the proposed method are considered for comparative analysis: (1) dimensionality reduction of the hybrid feature pool by PCA and then use of k-NN for classification, and (2) consideration of all the features (i.e., without using the feature selection technique) and then applying k-NN for classification. The comparative analysis results are listed in Table 5.

**Figure 6:**
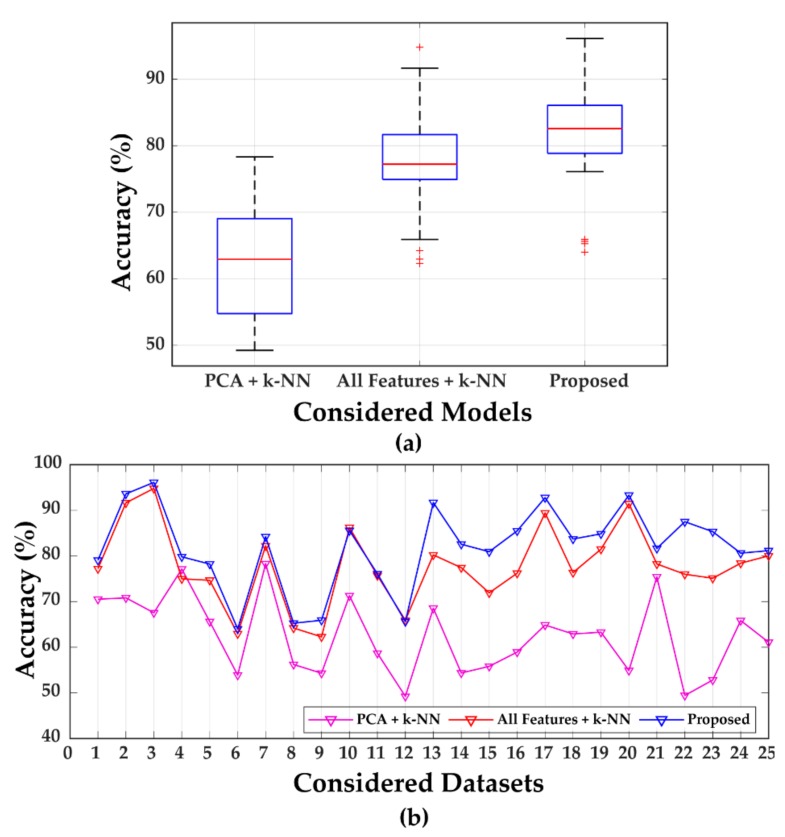
(**a**) Classification performance accuracy comparisons by boxplot, (**b**) performance chart based on accuracy comparisons.

Table 5 shows that PCA-based dimensionality reduction is unable to maintain the original information of the extracted hybrid feature pool. Therefore, the performance of classification, in this case, is relatively low. To prove the superiority of the proposed method, a one-way analysis of variance (ANOVA) test is performed on the classification results of the considered three methods. From the analysis of the variance, the obtained F-value is 34.20 and the *p*-value is 0. This indicates there is a significant difference between these three methods. Figure 6a depicts that among these three approaches, the proposed approach performs the best. There exist several outliers, as seen in the boxplot of Figure 6a, indicating that for a few datasets, the performance accuracy is low for the proposed method. However, from the line chart of Figure 6b, it can be visualized that the proposed approach still provides higher accuracies than the other two approaches for almost all the datasets described in Table 3 (Dataset 1 to 25).

Finally, to validate the robustness and superiority of the proposed approach, a comparison analysis is made after merging all the datasets numbered from 1 to 25 (from Table 3). Hence, the new dataset now contains data from all the experiment IDs. The proposed approach is compared with the other two approaches in the same manner as done previously for the individual datasets. Table 6 presents the comparative analysis results of different techniques for the new dataset.

## 4. Discussions

The experimental analyses provide insights for future research approaches. As can be seen in Table 3, the experimental ID for each individual participant is unique. Therefore, it is difficult to generalize the entire recorded response signals from all the participants for classifying stress and calm mental states. For example, if all the participants show a calm state for experimental IDs 4, 20, and 31, and a stress state for experimental IDs 1, 15, and 40, then it is easy to generalize the full dataset. Moreover, from Table 3, it is visible that the dataset is imbalanced in nature. Thus, SMOTE is adopted to handle this issue. Hence, the lack of proper preprocessing techniques (i.e., the preprocessed data from the DEAP dataset is directly considered) affects the classification performances of a few datasets. For example, in Table 4, datasets 6, 8, 9, and 12 give an accuracy rate of between 64%–66%, whereas the rest of the datasets give the accuracy within the range of 79%–96%. The performance degradation issues of datasets 6, 8, 9, and 12 are depicted in Figure 6a (several outliers are visible into the boxplot). Therefore, the performance of the proposed method can be further enhanced by using appropriate pre-processing techniques for EEG signals of different mental states. In addition to these, a comparative analysis of scalp sources can affect the final performance of the proposed approach. However, the focus of this research is to build an effective feature space that can represent the EEG signals in an accurate manner. The extraction of appropriate features from the EEG signals is highly needed for the construction of efficient classification models. As can be seen from Table 6, the Boruta-based k-NN classifier adopted in this paper outperformed the traditional k-NN classifier based on all features in terms of classification accuracy. Besides, the identification of electrodes and cortical regions containing the most relevant information can be investigated in the future to increase the performance of the proposed approach.

## 5. Conclusions

This paper proposes a mental state classification model based on whether or not a person is experiencing stress via the creation of a hybrid feature pool with the help of Boruta and the classification of instances using a k-NN classifier. For validation, final classification results were compared to conventional feature-driven k-NN approaches. When considering all features without subset selection from the hybrid feature pool, the k-NN method gave 69.26% accuracy, which is 4.12% lower than the proposed method. Compressing the feature pool with PCA gave an average of 65.62% accuracy, which is 7.76% lower than the proposed method. This suggests that the proposed model can better create distinct feature sub-sets from the designed hybrid feature pool for identifying the mental state of a person.

## Figures and Tables

**Table 1:** Mathematical description of the considered time-domain features.

Feature	Equation	Feature	Equation	Feature	Equation
F1	$\sqrt{\frac{1}{N}}\sum_{i=1}^{N}X_{i}^{2}$	F2	${\left(\frac{1}{N}\sum_{i=1}^{N}\sqrt{\left|X_{i}\right|}\right)}^{2}$	F3	max(|X|)−min(|X|)
F4	$\frac{1}{N}{\sum_{i=1}^{N}\left(\frac{X_{i}-\bar{X}}{\sigma }\right)}^{4}$	F5	$\frac{1}{N}{\sum_{i=1}^{N}\left(\frac{X_{i}-\bar{X}}{\sigma }\right)}^{3}$	F6	$\frac{F5}{F5^{4}}$
F7	$\frac{F5}{\frac{1}{N}\sum_{i=1}^{N}\left|X_{i}\right|}$	F8	$\frac{\max\left(\left|X\right|\right)}{F5}$	F9	$\frac{\max\left(\left|X\right|\right)}{\frac{1}{N}\sum_{i=1}^{N}\left|X_{i}\right|}$
F10	$\sqrt{\frac{\mathrm{var}\left(X\prime \right)}{\mathrm{var}\left(X\right)}}$	F11	$\frac{mobility\left(X\prime \right)}{mobility\left(X\right)}$		

*X* is the original EEG signal in the time domain. *N* denotes the total number of samples of the signal.

**Table 2:** Details of the correlated DWPT packets.

Frequency Bands	DWPT Packets	Presence in the Considered DEAP EEG Filtered Signals (4–45 Hz)	Usage of DWPT Packet
Delta	0–4 Hz	Not Present	No
Theta	4–8 Hz	Present	Yes
Alpha	8–12 Hz	Present	Yes
Beta	14–16 Hz	Present	Yes
	16–32 Hz	Present	Yes
Gamma	32–36 Hz	Present	No
	32–40 Hz	Present	Yes
	32–48 Hz	Not Present	No

**Table 3:** Details of the considered dataset.

Dataset	Participant ID	Considered Number of Channels	Experimental ID That Reflects the Distinctive State	
			Calm	Stress
**1**	**1**	32 EEG Channels	9,14	17,32,34,35,36,37
**2**	**2**		5,7,10,22,24,36	29,30,32,37,38
**3**	**4**		2,6,18	24,28,32
**4**	**5**		13,29	23,30,37
**5**	**8**		10,37,39	31,36
**6**	**10**		15,17,20,22,26,27,28	21,30,35,36,37,38,39
**7**	**11**		2,12,16,19,25,26,28,40	27,35,37,38,39
**8**	**12**		16,17,28	25,29,32,33,35,36,37,38
**9**	**13**		12,15,16	7,21,23,31,34,35,36,37,38,39
**10**	**14**		22,27	10,21,23,24,29,30,32,34,35,36,38
**11**	**15**		7,16,22,26	24,25,30,38
**12**	**16**		6,12,16,21,36	1,15,17,24,26,27,34
**13**	**18**		22,26,34	30
**14**	**19**		15,26,27	29,38
**15**	**20**		16,26,27,28,40	23,25,29
**16**	**21**		3,21,26,34,35	20,22,24
**17**	**22**		1,6,12,15,16,28	23,24,29,30,32,33,35,36,37,38,39
**18**	**24**		33,40	21,23,24,30,31,38,39
**19**	**25**		4,5,26,27,28,34	2,10,23,29,31,32,33,37,38,39
**20**	**26**		30	34
**21**	**27**		5,15,19,26,27,28,33,40	27
**22**	**28**		15,22,24,25	35,38
**23**	**29**		15,17	30,31,33,35
**24**	**31**		17,22,24,27,28,29	23,32,34,37,38,39
**25**	**32**		2,6,15,26,33	24,30,37

**Table 4:** Details of the class-wise and average classification accuracy.

Dataset	Participant ID	Class-Wise Accuracy (%)		Avg. Accuracy (%)
		Calm	Stress	
**1**	1	79.45	78.68	79.07
**2**	2	93.59	93.65	93.62
**3**	4	97.14	95.24	96.13
**4**	5	75	83.33	79.82
**5**	8	77.78	78.95	78.23
**6**	10	62.5	65.48	64.00
**7**	11	85.48	82.97	84.23
**8**	12	59.35	73.14	65.26
**9**	13	62.61	69.22	65.92
**10**	14	82.20	88.96	85.58
**11**	15	79.59	72.22	76.09
**12**	16	62.71	68.52	65.62
**13**	18	95.29	88.13	91.71
**14**	19	76	92.86	82.58
**15**	20	82.73	79.15	80.94
**16**	21	88.51	82.49	85.5
**17**	22	92.13	93.44	92.79
**18**	24	73.46	93.98	83.72
**19**	25	82.22	87.49	84.85
**20**	26	86.67	100	93.33
**21**	27	84.43	78.92	81.66
**22**	28	88.49	86.53	87.51
**23**	29	85.73	84.92	85.33
**24**	31	82.43	78.75	80.62
**25**	32	83.78	75.86	81.17

**Table 5:** Comparative analysis of different approaches for individual datasets.

Dataset	Participant ID	PCA + k-NN	All Features + k-NN	Proposed
**1**	1	70.52	77.22	79.07
**2**	2	70.83	91.65	93.62
**3**	4	67.56	94.81	96.13
**4**	5	77.14	75.0	79.82
**5**	8	65.62	74.67	78.23
**6**	10	53.89	62.95	64.00
**7**	11	78.32	82.22	84.23
**8**	12	56.22	64.21	65.26
**9**	13	54.31	62.28	65.92
**10**	14	71.29	86.23	85.58
**11**	15	58.68	75.71	76.09
**12**	16	49.23	65.91	65.62
**13**	18	68.55	80.24	91.71
**14**	19	54.36	77.44	82.58
**15**	20	55.81	71.92	80.94
**16**	21	58.94	76.22	85.5
**17**	22	64.87	89.44	92.79
**18**	24	62.91	76.36	83.72
**19**	25	63.30	81.49	84.85
**20**	26	54.89	91.45	93.33
**21**	27	75.43	78.25	81.66
**22**	28	49.43	76	87.51
**23**	29	52.82	75.12	85.33
**24**	31	65.85	78.42	80.62
**25**	32	61.09	80.03	81.17

**Table 6:** Comparative analysis of different approaches for the merged dataset.

Methods	Dataset	Avg. Accuracy (%)	Decrement from the Proposed Method (%)
PCA + k-NN	Merged dataset	65.62	7.76
All Features + k-NN		69.26	4.12
Proposed		73.38	-

The merged dataset is the finally formed dataset in which data from Dataset 1 to 25 (from Table 3) are considered together.
